# Boils in the Axillæ

**Published:** 1889-01

**Authors:** 


Boils in and about axillæ; scurfy, itching, moist herpetic eruption; pus continues to discharge from boils for an unusually long time, they no sooner heal than fresh ones appear—Lycopodium.

